# Editorial: Current perspectives in the diagnosis of parathyroid disease

**DOI:** 10.3389/fendo.2023.1323778

**Published:** 2023-10-30

**Authors:** Takahisa Hiramitsu, Zia Moinuddin, Titus Augustine

**Affiliations:** ^1^ Department of Transplant and Endocrine Surgery, Japanese Red Cross Aichi Medical Center Nagoya Daini Hospital, Nagoya, Japan; ^2^ Department of Transplant and Endocrine Surgery, Manchester Royal Infirmary, Manchester University Foundation Trust, Manchester, United Kingdom

**Keywords:** parathyroid disease, hyperparathyroidism, parathyroid gland, diagnosis, imaging modality, localization

Before performing a parathyroidectomy (PTx) for hyperparathyroidism (HPT), evaluating the localization of the parathyroid glands (PTGs) is essential. Conventional evaluation modalities include ultrasonography (US), computed tomography (CT), and technetium-99m methoxyisobutylisonitrile scintigraphy (^99m^Tc-MIBI). However, the diagnostic accuracy of these imaging modalities for multiglandular HPT is insufficient. Luo et al. evaluated a nomogram including US and clinical features for predicting multiglandular parathyroid disease (MPD) in primary HPT. The area under the receiver operating characteristic curve was 0.77. The specificity and sensitivity of a score of 65 points on the nomogram were 0.94 and 0.50, respectively. The diagnostic accuracy of this study was sufficient and suggests the possibility of a predictive model for MPD, although a limitation of this study was that it was not externally validated. The most common surgical approach for primary HPT is a minimally invasive unilateral approach. For preoperative surgical planning, the diagnosis of the number of causative PTGs may contribute to the decision regarding the surgical approach. This study may contribute to the prediction of MPD in primary HPT and preoperative surgical planning. Stoian et al. investigated the efficacy of bidimensional shear-wave elastography plane-wave ultrasound (2D SWE PLUS) in differentiating between thyroid nodules and PTGs. One reason for the insufficient diagnostic accuracy for the preoperative localization of PTGs on US and CT may be the difficulty in differentiating between thyroid nodules and PTGs. In this study, the diagnostic accuracy of 2D SWE PLUS for PTGs was demonstrated by comparing the elastic indices (EIs) of the normal thyroid tissue, thyroid nodules, and PTGs. The significantly different EIs between normal thyroid tissue and PTGs and between thyroid nodules and PTGs may contribute to differentiating PTGs from normal thyroid tissue and thyroid nodules. Significant differences in EIs between benign thyroid nodules and thyroid cancer and between PTGs in primary and secondary HPT have also been demonstrated. These findings may contribute to improving the diagnostic accuracy of preoperative localization of PTGs, as well as predicting the pathological diagnosis during preoperative evaluation. Further studies on the efficacy of 2D SWE PLUS in larger numbers of patients are warranted. Liu et al. investigated the efficacy of the maximum standardized uptake value (SUVmax) in fluorine-18 fluorocholine positron emission tomography/CT (^18^F-FCH PET/CT) for diagnosing PTGs. Conventionally, ^99m^Tc-MIBI is used for parathyroid scintigraphy. Recently, the effectiveness of ^18^F-FCH PET/CT in the diagnosis of PTGs has been reported. In this study, 60-min parathyroid/thyroid SUVmax on ^18^F-FCH PET/CT was the most effective parameter for diagnosing PTGs. Additionally, to differentiate parathyroid adenoma from hyperplasia, 60-min parathyroid SUVmax in ^18^F-FCH PET/CT is reported to be effective. This study also suggests the usefulness of ^8^F-FCH PET/CT for the preoperative localization of PTGs. Mokrysheva et al. has reported epidemiological research on primary HPT in the Russian Federation. This report highlights the importance of a national database, particularly for parathyroid diseases. Additionally, the detection rate of primary HPT has increased after the start of online registration. This may help advocate the importance of timely treatment. In this Research Topics, the recent diagnostic modalities and their usefulness were reported. We hope that this Research Topic will contribute to the development of research on parathyroid disease.

## Author contributions

TH: Writing – original draft. ZM: Validation, Writing – review & editing. TA: Supervision, Validation, Writing – review & editing.

